# Infectivity enhances prediction of viral cascades in Twitter

**DOI:** 10.1371/journal.pone.0214453

**Published:** 2019-04-17

**Authors:** Weihua Li, Skyler J. Cranmer, Zhiming Zheng, Peter J. Mucha

**Affiliations:** 1 LMIB, BDBC and School of Mathematics and Systems Science, Beihang University, Beijing, China; 2 Department of Computer Science, University College London, United Kingdom; 3 Systemic Risk Centre, London School of Economics and Political Sciences, London, United Kingdom; 4 Department of Political Science, The Ohio State University, Columbus, OH, United States of America; 5 Department of Mathematics, The University of North Carolina, Chapel Hill, NC, United States of America; West Pomeranian University of Technology, POLAND

## Abstract

Models of contagion dynamics, originally developed for infectious diseases, have proven relevant to the study of information, news, and political opinions in online social systems. Modelling diffusion processes and predicting viral information cascades are important problems in network science. Yet, many studies of information cascades neglect the variation in infectivity across different pieces of information. Here, we employ early-time observations of online cascades to estimate the infectivity of distinct pieces of information. Using simulations and data from real-world Twitter retweets, we demonstrate that these estimated infectivities can be used to improve predictions about the virality of an information cascade. Developing our simulations to mimic the real-world data, we consider the effect of the limited effective time for transmission of a cascade and demonstrate that a simple model of slow but non-negligible decay of the infectivity captures the essential properties of retweet distributions. These results demonstrate the interplay between the intrinsic infectivity of a tweet and the complex network environment within which it diffuses, strongly influencing the likelihood of becoming a viral cascade.

## Introduction

Massive data sets that comprehensively capture users’ behaviors in online social systems and their underlying network structures have reached an unprecedented scale, making it possible to develop computational methods to model complex patterns of human behavior at both individual and population levels [1–5]. Among various human-induced online processes, the study of social contagion—the spread of information, ideas, and behaviors through social networks—has attracted tremendous attention, especially in the fields of computational social science and network science [6, 7]. Many studies examine these peer-to-peer diffusion processes by focusing on a single piece of information and making assumptions about infectivity, recovery probabilities, and their intrinsic relations to network structures [4, 8–12]. We consider measuring the infectivity of information cascades to be the crux for predicting their ultimate virality.

Previous research has successfully advanced the modelling of information spread by studying memes in Twitter data, where a meme is defined by the use of a hashtag and includes all of the tweets with that hashtag [5, 13–16]. Gleeson et al. introduced a mathematical framework to examine the branching dynamics of this meme spread process [17]. Besides these theoretical efforts, many other studies try to explore this research topic with large scale empirical data and real world experiments. Vosoughi et al. analyzed over ten years of Twitter data on the dynamical diffusion of true and false news [12]. Bail et al. ran a field experiment on Twitter to study the spread of views and political opinion [18]. Del Vicario et al. studied emotional contagion and group polarization on another popular online social network platform: Facebook [19]. All these efforts provide a deeper understanding of social factors and behavioral patterns in online information spread.

**Fig 1:**
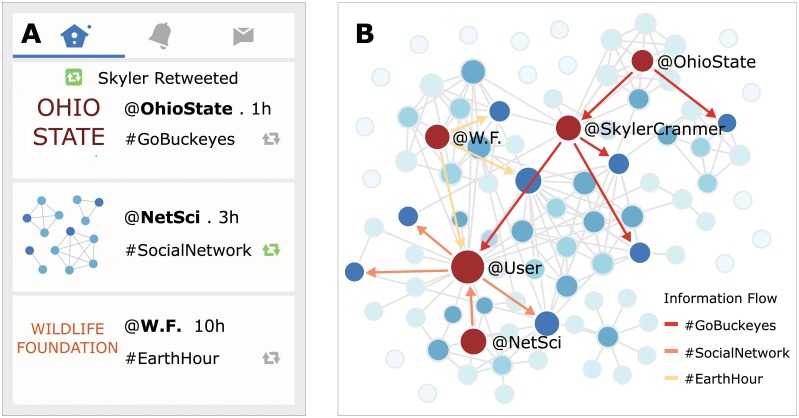
Schematic of social contagion information diffusion in Twitter. **(a)** The Twitter user interface that displays three latest tweets with different degrees of interestingness from her friends. The first message was originally posted by someone with whom she does not have direct connection, but she is still able to see it after being retweeted by one of her friends. She chose to retweet the second tweet she found interesting, extending the information flow of that message to all her followers. If the “memory length” of this user is 3, she will not read or retweet messages posted more that 10 hours ago (the time of the third item in the display). **(b)**, The online network environment of involved users and the flows of information cascades.

Here, we reanalyze these data with an exclusive focus on modelling the direct transmission of information through a social network in the form of retweets. Our reason for focusing on retweets is that the transmission of a particular hashtag is more likely to occur not only from person to person through online social ties [15], but also through mass targeted broadcasting from other media sources outside the specific social network. As observed in Ref. [20], broadcasts contribute substantively to viral events, e.g., the World Cup Final attracts about 1 billion viewers worldwide, while news coverage from popular websites also reaches a similar number of Internet users. In such popular events, the discussion of a meme in broadcasting media (e.g. social network platforms, TV shows, radio and news reports) can greatly boost its spread. Retweets, by contrast, constitute an information cascade that originated from an identifiable individual user and is a contagion spread mostly through the links of the follower network (Fig 1).

## Materials and methods

### Data

The Twitter data, studied previously in Refs. [14] and [15], comprise a reciprocal follower network of *N* = 595, 460 nodes and the time-stamp record of *N*_*twt*_ = 12, 054, 205 tweets, of which *N*_*ret*_ = 1, 687, 704 are retweets, within a total time frame of *T* = 33 days and we treat a day as the time step. According to Refs. [14] and [15], they complied with the terms of service for Twitter from which they collected data and the data were analysed anonymously. The data were collected in three data sets: (1), a reciprocal follower network where each edge is a pair of Twitter users who are following each other; (2), tweet timeline data with the hashtags and their adopters sorted by timestamp; (3), the retweet timeline data where each line is a hashtag followed by the sequence of its adopters retweeting about this hashtag from other users sorted by timestamp. Note that the retweet data set is a subset of the tweet data set.

### Generating functions

The modeling of human factors—specifically a dynamical process with limited user memory length—can help to unveil the core features of contagion in complex social systems driven by peer-to-peer influence. At every time step, a user generates a new tweet with innovation probability *β* = (*N*_*twt*_ − *N*_*ret*_)/*NT*. The infectivity λ_0_ of a cascade is the probability that a follower will retweet it in one time step. Let us consider the dynamical process of retweeting in more detail by focusing on a given information cascade with infectivity λ_0_, posted online at time *t* = 0, assuming for simplicity that all other cascades have infectivity equal to its mean, 〈λ_0_〉. We denote the distribution of retweets at time *t* by *q*_*n*_(*t*), which is the probability that a cascade has popularity *n* at *t*. Following the probability generating function (PGF) formalism in Refs. [17, 21, 22], we define the cascade PGF, parameterized by *x*, to be $H\left(t,x\right)\equiv \sum_{n=1}^{\infty }q_{n}\left(t\right)x^{n}$. We assume the in-degree of all nodes to be 〈*k*〉, and characterize the heterogeneity of the out-degree distribution with PGF $f\left(x\right)\equiv \sum_{k=0}^{\infty }p_{k}x^{k}$, where *p*_*k*_ is the probability of a node with out-degree *k*. We seek to quantify *G*(*t*, *x*) as the PGF for the retweet distribution at time *t* of a random cascade branch that originates from a single user randomly chosen from a given cascade. For the user and all of her followers, a tweet event increases the popularity of the given cascade by 1, and places it at the top of the memory length window. As a result, the PGF for the number of tweets at time *t* is given by [17] *H*(*t*, *x*) = *xG*(*t*, *x*)*f*(*G*(*t*, *x*)). Denoting the rate of a user’s tweet activity as *ρ* = (*β*(〈*k*〉 + 1) + 〈λ_0_〉〈*k*〉*M*)/*M*, and following the analysis from Ref. [17], the differential equation for *G*(*t*, *x*) is obtained:

$$\begin{array}{c} \frac{\partial G}{\partial t}=\lambda_{0}xf\left(G\right)+\rho -\left(\lambda_{0}+\rho \right)G, \end{array}$$ (1)

which can be solved with initial conditions *f*′(1) = 〈*k*〉 and *G*(0, *x*) = 1.

The above PGF provides a prediction of the expected popularity *m*(*t*) for the focal tweet at time *t*, and by definition the number of retweets is *m*(*t*) − 1. In the case of constant infectivity with no decay effect, Eq (1) leads to

$$\begin{array}{c} m\left(t\right)=\left(2\lambda_{0}+\rho \right)\tau +\left(1-\left(2\lambda_{0}+\rho \right)\tau \right)\text{exp}(-t/\tau ), \end{array}$$ (2)

where *τ* ≡ 1/(*ρ* − λ_0_(〈*k*〉 − 1)). When λ_0_ is small enough such that *τ* > 0, Eq (2) suggests that the popularity converges to a finite level. In contrast, for λ_0_ large enough and *τ* < 0, Eq (2) indicates that popularity grows exponentially with time. The threshold separating these two behaviors is at

$$\begin{array}{c} \bar{\lambda_{0}}=\rho /\left(\left\langle k\right\rangle -1\right). \end{array}$$ (3)

Above this threshold, information can spread to a global scale; However, when *t* → ∞ the exponential growth prediction *m*(*t*) → ∞ does not conform with real data, calling for additional effects to reproduce the empirical process.

### Decay factor and infectivity estimation

Previous studies have found that the attractiveness of online information does not remain constant over an indefinite period of time, but rather gradually declines as it grows older [23]. We adopt this observation of fading popularity by incorporating a decay factor *α* and assume that the infectivity of cascade *i* decays exponentially by λ_*i*_(*t*) = λ_*i*0_*e*^−*α*(*t*−*t*_*i*0_)^, where *t*_*i*0_ is the time of the initial tweet. Among retweets for which we can identify at least one of the previous tweets in the same cascade posted by their neighbors, a fraction *ψ* = 0.69 of them occurred within one day after the tweet was last seen by the retweeted user. Using a mean-field approach that assumes the degree of all nodes to be equal to 〈*k*〉, we then express the average number of retweets of cascade *i* at time *t* as *a*_*i*,*t*_ = λ_*i*0_*e*^−*αt*^〈*k*〉*a*_*i*,*t*−1_/*ψ*.

We define the number of total retweets of cascade *i* at time *t* as *A*_*i*,*t*_, and derive the conditional expectation of *A*_*i*,*t*_ given that cascade *i* is retweeted at least once during its lifetime:

$$\begin{array}{c} E\left(A_{i,t}|A_{i,t}\geq 1\right)\equiv \sum_{\tau =1}^{t}a_{i,\tau }=\sum_{\tau =1}^{t}{\left(\frac{\lambda_{i0}\left\langle k\right\rangle }{\psi }\right)}^{\tau }e^{-\frac{1}{2}\alpha \tau \left(\tau +1\right)}. \end{array}$$ (4)

Here we make two assumptions about the retweet size and infectivity of cascades: first, the tweet will either be stifled by stochastic fluctuations at the beginning such that no followers retweet it, or will be retweeted with probability 〈*k*〉λ_*i*_(*t*)*ψ*^−1^ and reach the mean size determined by Eq (4) at time *t*; second, for fixed values of *t* and *A*_*i*,*t*_, the infectivity λ_*i*0_ calculated by Eq (4) is the minimum rate to reach a retweet size ≥ *A*_*i*,*t*_. We further assume that the relation between the number of retweets *S*_*i*_ in the real Twitter data and *A*_*i*,*t*_ is *S*_*i*_ = *A*_*i*,*t*_|_*t*→∞_. Then we set *t* = 25 to fit the spread rate distribution in Eq (4). As such, we can obtain (λ_*i*0_, *S*_*i*_) pairs such that their probability distribution satisfies *P*(*S* ≥ *S*_*i*_) = *P*(λ_0_ ≥ λ_*i*0_), which can be used to approximately estimate the distribution of λ_0_ from empirical Twitter data.

The above analysis has taken the decay effect into account. We next approximate the distribution of initial infectivity λ_*i*0_ for cascade *i* as a truncated lognormal form with an upper bound probability λ_max_. Let *p*^0^(λ_0_) be the lognormal distribution $p^{0}\left(\lambda_{0}\right)={\left(\lambda_{0}\sigma \sqrt{2\pi }\right)}^{-1}e^{-{\left(\text{ln}\lambda_{0}-\mu \right)}^{2}/2\sigma^{2}}$, where *μ* and *σ* are parameters, and the normalization factor for the infectivity distribution can be written as $P^{0}\left(\lambda_{\text{max}}\right)=\int_{0}^{\lambda_{\text{max}}}p^{0}\left(\lambda_{0}\right)d\lambda_{0}$. Thus we have the probability distribution of infectivity *p*^infectivity^(λ_0_) = *p*^0^(λ_0_)/*P*^0^(λ_max_) in the truncated lognormal form with 0 < λ_0_ < λ_max_. If a random user tweets a cascade with initial infectivity λ_0_, and it stays in the followers’ memory for an average lifetime 1/*ψ*, the probability that it is not retweeted by any follower is (1 − λ_0_)^〈*k*〉/*ψ*^. Therefore, the fraction of cascades being retweeted at least once is given by

$$\begin{array}{c} P\left(\lambda_{0}\right)=\int_{0}^{\lambda_{0}}p^{\text{infectivity}}\left(\tau \right)\left[1-{\left(1-\tau \right)}^{\langle k\rangle /\psi }\right]d\tau . \end{array}$$ (5)

**Fig 2:**
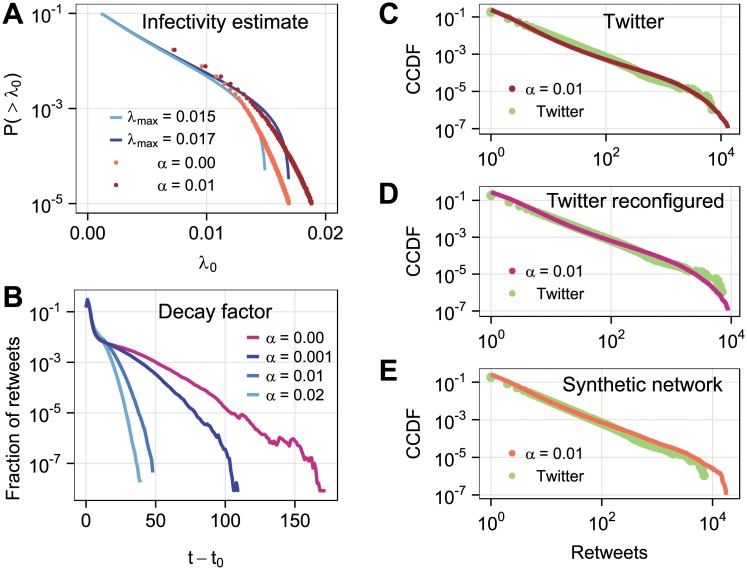
Simulation parameter settings and results. **a**, Truncated lognormal fit. Light and dark blue lines are fit with theoretical distribution function Eq (6), and the red and orange points are fit with distribution computed from real Twitter data with Eq (4). The parameters used in Eq (6) are as follows: when decay factor *α* = 0, *μ* = ln 0.0012, *σ* = ln 2.4, λ_max_ = 0.015; when *α* = 0.01, *μ* = ln 0.0012, *σ* = ln 2.4, λ_max_ = 0.017. **b**, Retweets at time *t* of cascades originated at time *t*_0_ with different decay factors. **c-e**, Complementary cumulative distribution functions (CCDFs)—the fraction of cascades with more than *n* retweets for numerical simulations, compared with retweets from empirical Twitter data marked by green points. The model parameters are identical except for the network structure: **c**, The empirical Twitter follower network with *N* = 5.95 × 10^5^ and 〈*k*〉 = 47.94; **d**, Reconfiguration of the empirical Twitter network preserving the degree distribution; **e**, Scale-free network with *N* = 5 × 10^5^, 〈*k*〉 = 48 and exponent *γ* = 2.8.

This expression captures the fact that information cascades are likely to be stifled due to stochastic fluctuations at the initial stage, before it actually starts spreading. Assuming the infectivity is small such that [1 − (1 − λ_0_)^〈*k*〉/*ψ*^] ≃ λ_0_〈*k*〉/*ψ*, we have

$$P(\lambda_{0})=\frac{\left\langle k\right\rangle }{2\psi P^{0}\left(\lambda_{\text{max}}\right)}e^{\mu +\frac{\sigma^{2}}{2}}[1+\text{erf}(\frac{\text{ln}\lambda_{0}-\mu -\sigma^{2}}{\sigma \sqrt{2}})],$$ (6)

where erf(*x*) is the error function. We then estimate (λ_*i*0_, *S*_*i*_) pairs from empirical data with a pre-assumed decay factor *α* from Eq 4, and fit the outcome distribution with Eq 6 (see Fig 2a).

### Simulations

The simulations start with a set of users generating tweets, the infectivity of which follow a truncated lognormal probability distribution, with a universal decay factor governing their long time dynamics. When a user tweets a new message by herself, or retweets an old message from her followees, illustrated in Fig 1, all of her followers will receive the message. A user will only see the latest tweets within her memory length, which is a fixed value for all users [13, 16, 24, 25]. A natural measure of popularity is the number of retweets plus one that accounts for the original tweet, and we regard each not-retweeted tweet as a cascade with popularity 1. The innovation probability (the probability that a user generates a brand new tweet) *β* = 0.528 is calculated from Twitter data.

The mean degree of the Twitter follower network is 〈*k*〉 = 47.94 with a total number of *N*_*cas*_ = *N*_*twt*_ − *N*_*ret*_ = 10, 366, 501 cascades, of which 962, 341 are cascades with popularity > 1. Each time step a user retweets or creates on average *N*_*twt*_/*NT* cascades that will be retweeted 〈λ〉〈*k*〉*N*_*twt*_/*NT* times by her followers in the next time step, leading to an estimate of average infectivity as 〈λ〉 = *ψN*_*ret*_/〈*k*〉*N*_*twt*_ = 0.002. Memory length can thus be estimated by *M* = *ψN*_*ret*_/〈λ〉*NT* = 43, and the threshold $\bar{\lambda }$ in Eq (3) is 0.015. We use decay parameter *α* = 0.01, and the corresponding infectivity distribution parameterized by *μ* = ln 0.0012, *σ* = ln 2.4 and λ_max_ = 0.017 to obtain the blue curve in Fig 2a fitting to the red dots of (λ_*i*0_, *S*_*i*_) pairs calculated from Twitter data.

In all simulations, we first run a burn-in period of 100 time steps. As the Twitter data focus on new memes, we only analyse new cascades that originate in the next *T* = 33 time steps.

## Results

The Twitter data we use contains a follower network with 6.0 × 10^5^ users, 1.7 × 10^6^ retweets and 1.2 × 10^7^ tweets generated by these users in 33 days [14, 15]. We estimate the probability distribution of the infectivity of cascades from real data, and simulate the process on the follower network (see Methods). A cascade consists of retweets that have the same hashtag and the same user who initially posted the tweet, together with the tweet that originated the cascade.

Previous studies have demonstrated that the topology of networks, especially the community structure, has pronounced effects on information diffusion [14, 26]. Communities could promote spread by homophily and social reinforcement, but may also hinder wider spread by trapping information, resulting in a high concentration of retweets within a community. To examine the influence of community structures, Weng et al. [14] introduced two statistical features of memes, which we modify for retweet cascades: the adoption dominance *g* computes the proportion of users retweeting the cascade in the community with the most adopters; and the retweet entropy *H*^*r*^ quantifies the distribution of retweets across different communities, as a measure of the concentration of the cascade across communities. We compute both measures based only on retweets in their early stages (first 50 tweets) to avoid bias from a cascade’s popularity.

**Fig 3:**
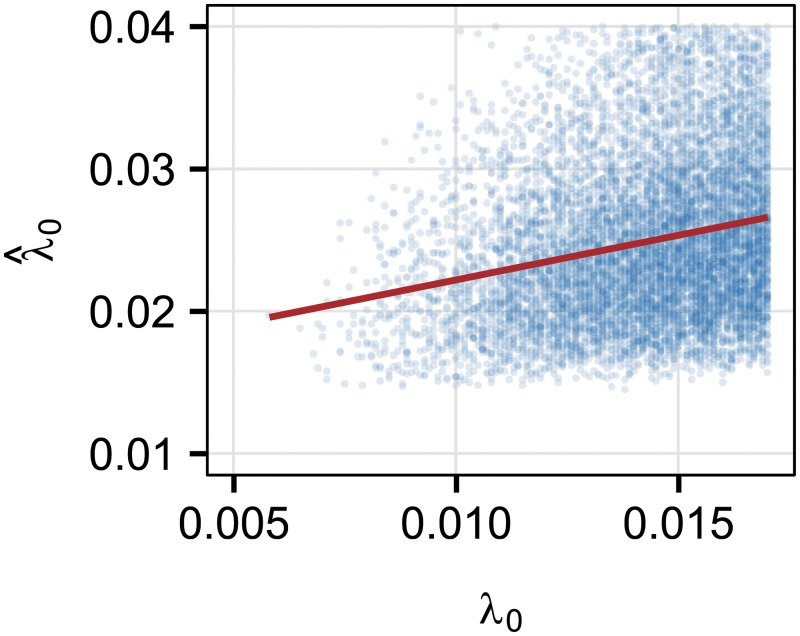
Comparison between real and estimated infectivity in simulations. Real infectivity λ_0_ and estimated infectivity ${\hat{\lambda }}_{0}$ computed from simulation data according to Eq 2 without considering the decay effects. The solid line is the linear regression fit. Estimates are calculated from only the first 50 retweets of each tweet, so that they may be used to try to predict whether a given cascade “goes viral”.

Retweet cascades are very different from hashtag memes in that we can more realistically assume that social contagion through the follower network is the major mechanism by which the retweet cascade is propagating. To provide direct evidence of this, we sampled 10^5^ tweets and retweets, respectively, finding that for 23.8% of tweets we can find at least one earlier tweet with the same hashtag from the user’s friends, while 46.0% of retweets have at least one friend who previously retweeted in the same cascade. Importantly, these percentages are limited by the specific follower network available in the data set, which inherently undercounts the possibility of transmission through the online social network because the network in the data only includes the reciprocal following ties (to better reflect real social relations). We estimate the infectivity of a specific cascade assuming that all such identifications are the actual paths of information transmission, using only the first 50 retweets (see Methods). Despite the relatively high inaccuracies observed between the true and predicted infectivities in our simulated data (where we know the true imposed infectivity, cf. real Twitter data), we note the overall trends of the infectivity estimates are in the right direction, with a slope of 0.92, *R*^2^ = 0.05 and p-value < 0.01 (Fig 3). The distribution of estimated infectivity is heavy tailed and not Gaussian, and the *R*^2^ of the linear model is low. Interestingly though, as we proceed to consider predictive models for virality that include such estimates of cascade infectivity, we will see that it improves prediction despite relatively poor fit.

We now test whether this simple model of infectivity demonstrates predictive power for identifying viral retweet cascades in real Twitter data. In Ref. [14], Weng et al. used community concentration features to predict viral memes with three models: the random guess (RG) model randomly samples the cascade without any predictors; the null model (NM, referred to as the “community-blind model” in Ref. [14]) employs the number of distinct users and the total number of neighbors of early retweet users; the community-based (CB) model also incorporates three community-based features of the Twitter network: the number of infected communities, retweet entropy *H*^*r*^, and the fraction of intra-community user interactions. We introduce two additional models adding features to the NM model to predict viral cascades with infectivity estimates: the infectivity-based (IB) model uses the estimated rate of infectivity $\hat{\lambda_{0}}$ from Eq (2), where 〈*k*〉 is the mean degree of early retweet users; and the community & infectivity based (C&I) model combines all of these infectivity and community-based features. Each of our classifiers includes only information about the first 50 retweets of each tweet, to try to predict whether the retweet cascade “goes viral”. We train random forest classifiers on 1, 272 real Twitter cascades and 20, 000 simulated cascades sampled from 20 replications, using 10-fold cross validation to predict viral cascades that attract more retweets than a certain percentile threshold *θ* of all cascades.

**Fig 4:**
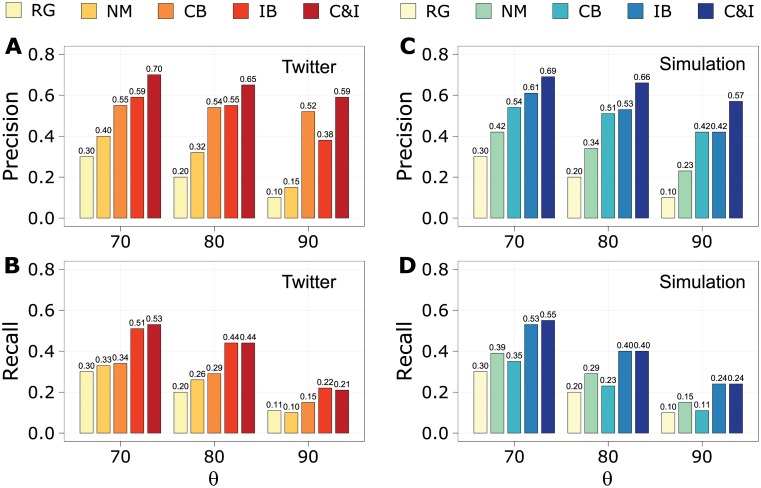
Random forest model predictions. We predict whether a cascade will go viral or not; a cascade is viral if it produces more retweets than a certain percentile threshold (*θ* = 70, 80, 90) of cascades, using community-based features and infectivity estimates that are calculated based on the initial 50 retweets for each cascade. Random forests are trained on sets of features delineated by the labels RG, NM, CB, IB and C&I (see the main text). The classifier including estimated infectivity (IB) typically outperforms the community-based model (CB), while combining all of the community-based and infectivity features (C&I) gives the best predictions overall. **a**, Precision rates of Twitter data. **b**, Recall rates of Twitter data. **c**, Precision rates of retweet data from simulations. **d**, Recall rates of retweet data from simulations. The precision and recall rates reported in this figure are mean values of 100 randomizations of the random forest model.

The results on the Twitter data suggest that in most cases our IB model performs better than the CB model (Fig 4a and 4b), indicating that estimated infectivity alone can improve the prediction even more than the community-based predictors. Moreover, the C&I model, incorporating both community and infectivity factors, reveals a striking increase of predictive power above the other models. Fig 4c and 4d shows random forest model prediction and recall rates on retweet data generated by our simulations, indicating patterns consistent with those observed in the Twitter data. The IB model, only adding infectivity to the NM model, is comparable to the CB model that includes three community features, and by considering all predictors the C&I model excels in both precision and recall rates. We note that replacing the estimated ${\hat{\lambda }}_{0}$ by the true λ_0_ used in the simulations—a test we can obviously not reproduce in the real Twitter data—yields additional improvement in classification (S2 Table), suggesting substantial potential for a more refined estimate of $\hat{\lambda_{0}}$ to lead to even greater accuracy for predicting viral cascades.

We further test our results using logistic regression with the same set of features as in the C&I model. We find that estimated infectivity is still a significant predictor in simulation data, but not in predicting virality in the real Twitter data (S3–S5 Tables). There may be multiple reasons for this apparent discrepancy between the random forest and logistic regression results. One possibility is that logistic regression is too specific in the functional form in which it estimates the probability of virality. In particular, we note the substantial noise in estimating infectivity we observe in our simulations; without any way to compare the estimated infectivities with “true” values in the real Twitter data, we cannot know whether the effect of this noise interacts poorly with the log-odds-shift assumptions of logistic regression.

Our simulations emulate the real-world diffusion process in Twitter by taking into consideration several human behavioral factors, such as a limited memory length and a gradual decrease in interest, in a simplified simulation model. We estimate a fixed memory length for all users from data and additionally incorporate a small but non-zero decay parameter to the infectivity of each cascade (see Methods). The initial infectivities of cascades are sampled from a probability distribution computed from empirical data (Fig 2a). The decay effect mainly affects the long time dynamics of viral cascades (Fig 2b). If we ignore the decay effect of infectivity, cascades with large infectivity will still keep spreading after long periods of time, even with fixed user memory length. With a small but non-zero decay parameter *α*, even the most popular cascades will diminish at some point, and the system quickly reaches equilibrium. We then use simulations on networks with different structural properties but otherwise identical parameter settings to calculate the distributions of cascade sizes.

Fig 2c shows that our simulations on the Twitter follower network replicate well the cascade distribution observed in the data. We also run a simulation on a configuration model network with the same degree distribution as the empirical Twitter network (Fig 2d). Simulation results on a synthetic network generated by the algorithm in Ref. [27] with the power-law exponent *γ* = 2.8, representing an analogous degree heterogeneity of the Twitter network (see S3 Fig), also recover the statistical features of Twitter data (Fig 2e). When we switch the decay parameter to 0.001 and 0.02, respectively, we still replicate the empirical retweet distribution fairly well by changing the corresponding λ_max_ parameter (S5 Fig).

## Discussion

We have demonstrated the predictive power of infectivity for identifying viral retweet cascades in real-world Twitter data and in simulation. An important assumption of this study is that the spread of retweet cascades resembles the peer-to-peer social contagion through the Twitter follower network, which we argue is different from viral memes represented by hashtags that more heavily rely on transmission through broadcasting. We demonstrate that the early spread rate for retweet cascades can be a good indicator of the intrinsic interestingness of a tweet, and that the corresponding estimate of infectivity gives improved prediction of virality. But, importantly, the same scheme might not readily apply to some memes that need to be broadly broadcast before they become viral. This difference may help explain why the measure of early infectivity of a hashtag in Ref. [15] does little to improve the prediction of viral memes.

Our mean-field method to estimate infectivity from empirical data clearly leaves plenty of room for improvement. The predictive ability of machine learning methods improves further on simulation data when we include the true infectivity, demonstrating the importance of accurate estimations of the cascade infectivity. Apart from this indirect approach with strong assumptions, we could also design a more straightforward method. The biggest challenge for such a measurement is to gather large-scale, high-quality data with which it is possible to infer accurate retweet relations. Better data and more reliable methodology to estimate infectivity are key to improving the predictive power.

Our study shows that infectivity improves the prediction of viral cascades that are mostly induced by contagion along the links representing social network connections. Network community structure captures additional local environmental factors such as homophily, social reinforcement and a trapping effect that further affect the spread and likelihood of virality of retweet cascades. Nevertheless, the infectivity determines the internal attractiveness and seems to be one of the most important factors in driving the virality of a cascade. Said another way, we have successfully demonstrated that the inherent quality of content—in the sense of being sufficiently interesting to have high infectivity—is an essential element promoting the chances of a successful spread that might not otherwise be as plausible in light of the local environmental factors.

## Supporting information

S1 Fig Distribution of tweets and retweets in Twitter data. (PDF)

S2 Fig Real and estimated infectivity distributions. (PDF)

S3 Fig Degree distribution of networks. (PDF)

S4 Fig Lognormal distribution fit with different decay parameters. (PDF)

S5 Fig Simulation on Twitter follower network and other synthetic networks. (PDF)

S6 Fig Statistics based on community structure. (PDF)

S1 Table Statistics of networks used in simulation models. (PDF)

S2 Table Random forests results in 10-fold cross validation. (PDF)

S3 TableLogistic models of viral cascade prediction in Twitter data with estimated infectivity ${\hat{\lambda }}_{0}$.(PDF)

S4 TableLogistic models of viral cascade prediction in simulation data with estimated infectivity ${\hat{\lambda }}_{0}$.(PDF)

S5 Table Logistic models of viral cascade prediction in simulation data with true infectivity λ_0_. (PDF)

S1 File Supporting information. (PDF)
