# Supplementary material for: Infectivity enhances prediction of viral cascades in Twitter
Source: PLoS One. 2019 Apr 17;14(4):e0214453. doi: 10.1371/journal.pone.0214453 (PMC6469756; doi:10.1371/journal.pone.0214453)
Supplement: S1 Fig — (PDF) [file pone.0214453.s001.pdf]

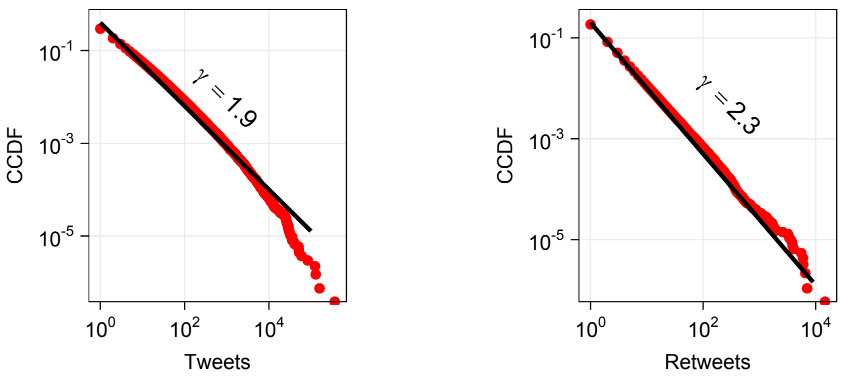

Fig 1. Distribution of tweets and retweets in Twitter data. *(Left)*: complementary cumulative probability distribution (CCDF) of hashtags (memes); *(Right)*: CCDF of cascades. Black lines indicate power laws with given exponents γ.
