# Supplementary material for: Infectivity enhances prediction of viral cascades in Twitter
Source: PLoS One. 2019 Apr 17;14(4):e0214453. doi: 10.1371/journal.pone.0214453 (PMC6469756; doi:10.1371/journal.pone.0214453)
Supplement: S2 Fig — (PDF) [file pone.0214453.s002.pdf]

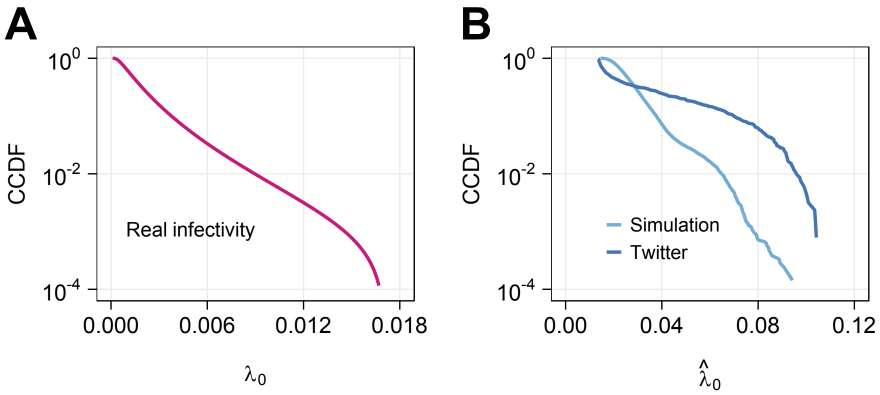

Fig 2. Real and estimated infectivity distributions. **a**, Real infectivity distribution used for simulation models with $\mu = \log 0.0012$, $\sigma = \log 2.4$, $\lambda_{\max} = 0.017$. **b**, Estimated infectivity based on the first 50 retweets in simulation and Twitter retweet data.
