# Supplementary material for: Infectivity enhances prediction of viral cascades in Twitter
Source: PLoS One. 2019 Apr 17;14(4):e0214453. doi: 10.1371/journal.pone.0214453 (PMC6469756; doi:10.1371/journal.pone.0214453)
Supplement: S3 Fig — (PDF) [file pone.0214453.s003.pdf]

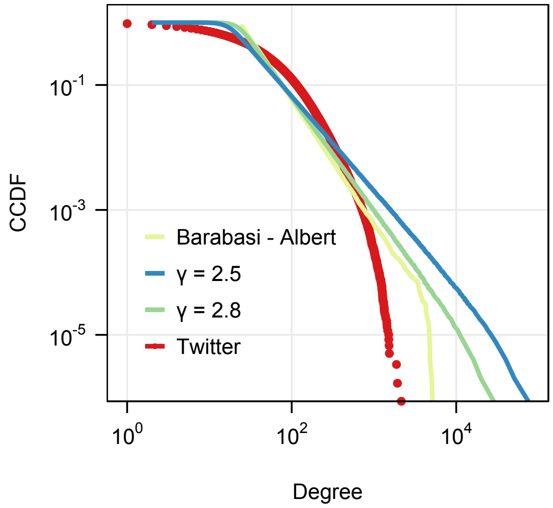

Fig 3. Degree distribution of networks. The degree distribution of the undirected Twitter follower network of reciprocal ties, and other synthetic networks used in our simulations. We later show that when a synthetic network has a similar degree distribution to the Twitter network, we could reproduce the cascade size distribution of retweets.
