# Supplementary material for: Infectivity enhances prediction of viral cascades in Twitter
Source: PLoS One. 2019 Apr 17;14(4):e0214453. doi: 10.1371/journal.pone.0214453 (PMC6469756; doi:10.1371/journal.pone.0214453)
Supplement: S4 Fig — (PDF) [file pone.0214453.s004.pdf]

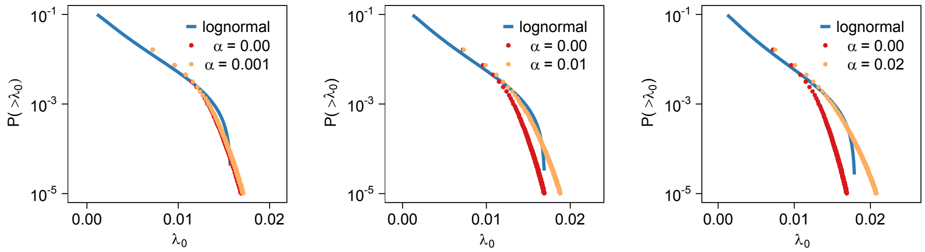

Fig 4. Lognormal distribution fit with different decay parameters. (*Left*): $\alpha = 0.001$ and $\lambda_{\max} = 0.0158$; (*Middle*): $\alpha = 0.01$ and $\lambda_{\max} = 0.017$; (*Right*): $\alpha = 0.02$ and $\lambda_{\max} = 0.018$. For all panels the other parameters are the same as those in the main text: $\mu = \log 0.0012$ and $\sigma = \log 2.4$.
