# Supplementary material for: Infectivity enhances prediction of viral cascades in Twitter
Source: PLoS One. 2019 Apr 17;14(4):e0214453. doi: 10.1371/journal.pone.0214453 (PMC6469756; doi:10.1371/journal.pone.0214453)
Supplement: S5 Fig — (PDF) [file pone.0214453.s005.pdf]

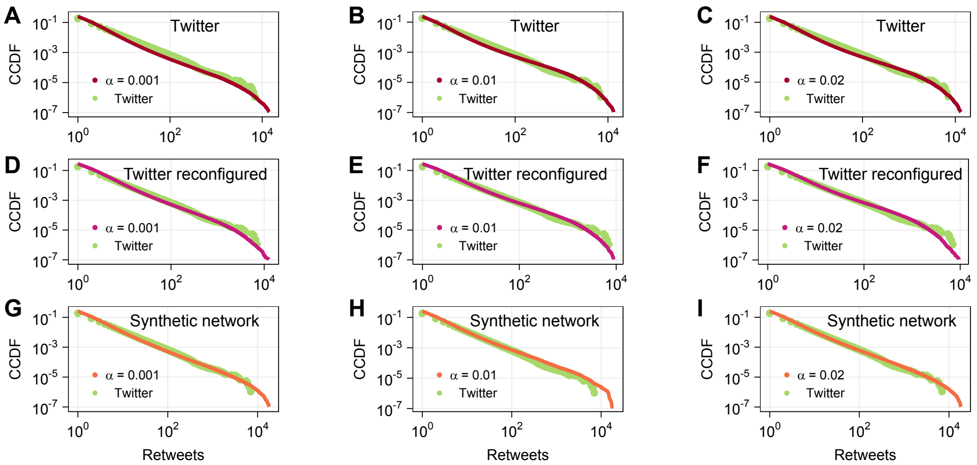

Fig 5. Simulation on Twitter follower network and other synthetic networks. Parameter settings are: (*Left panel*): $\alpha = 0.001, \lambda_{\max} = 0.0158$; (*Middle panel*): $\alpha = 0.01, \lambda_{\max} = 0.017$; (*Right panel*): $\alpha = 0.02\lambda_{\max} = 0.018$. Other parameters: $\mu = \ln 0.0012, \sigma = \ln 2.4, M = 43, \beta = 0.528$. For the synthetic networks, the power-law exponent is $\gamma = 2.8$ and mean degree $\langle k \rangle = 48$. We ran $1,000$ simulations for each plot.
