# Supplementary material for: Infectivity enhances prediction of viral cascades in Twitter
Source: PLoS One. 2019 Apr 17;14(4):e0214453. doi: 10.1371/journal.pone.0214453 (PMC6469756; doi:10.1371/journal.pone.0214453)
Supplement: S6 Fig — (PDF) [file pone.0214453.s006.pdf]

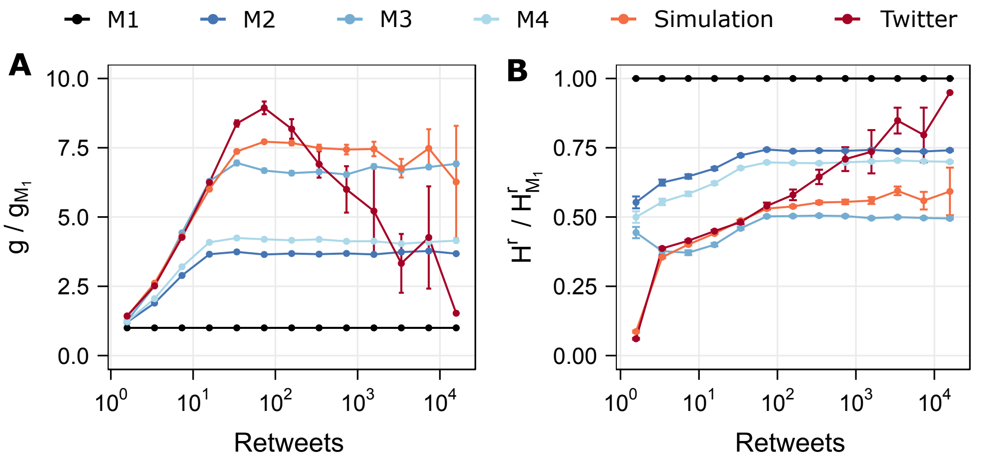

Fig 6. Statistics based on community structure. **a**, Adoption dominance g for baseline models M1, M2, M3, M4, Twitter retweet data and the simulation retweet data of attention limit model based on the first 50 retweets. **b**, Retweet entropy H^r for baseline models M1, M2, M3, M4, Twitter retweet data and the simulation retweet data of attention limit model based on the first 50 retweets. Our simulation model is closest to real Twitter data statistics.
