# Supplementary material for: Infectivity enhances prediction of viral cascades in Twitter
Source: PLoS One. 2019 Apr 17;14(4):e0214453. doi: 10.1371/journal.pone.0214453 (PMC6469756; doi:10.1371/journal.pone.0214453)
Supplement: S1 Table — (PDF) [file pone.0214453.s007.pdf]

Table 1. Statistics of networks used in simulation models. We present here the detailed network statistics of the Twitter follower network, the Barabasi - Albert network, synthetic networks in Ref. 8 with exponent $\gamma = 2.8$ and $\gamma = 2.5$.

Network	N	$\langle k \rangle$	$\langle k^2 \rangle$	$k_{\max}$	$\langle k^2 \rangle / \langle k \rangle$
Twitter	5.95×10^5	47.94	7.29×10^3	2.15×10^3	1.52×10^2
Barabasi - Albert	5×10^5	48	7×10^3	5×10^3	1.5×10^2
Synthetic network $\gamma = 2.8$	5×10^5	48	1×10^4	3×10^4	3×10^2
Synthetic network $\gamma = 2.5$	5×10^5	48	5×10^4	7×10^4	1×10^3
