# Supplementary material for: Infectivity enhances prediction of viral cascades in Twitter
Source: PLoS One. 2019 Apr 17;14(4):e0214453. doi: 10.1371/journal.pone.0214453 (PMC6469756; doi:10.1371/journal.pone.0214453)
Supplement: S2 Table — (PDF) [file pone.0214453.s008.pdf]

Table 2. Random forests results in 10-fold cross validation. Cascades with size $S \geq 50$ are considered. Simulated retweet data are generated with $\alpha = 0.01$ from 20 replications. All models in this table use the two null model predictors: The number of distinct users, and the total number of neighbors of early retweet users. There are three community–based predictors: The number of infected communities, user entropy H^u, and the fraction of intra–community user interactions. $\hat{\lambda_0}$ is the infectivity estimated by equation (2) without accounting for the decay factor. λ_0 is the real infectivity used in simulations.

Model	Data	$\hat{\lambda_0}$	λ_0	Community	$\theta = 90\%$		$\theta = 80\%$		$\theta = 70\%$	
					Precision	Recall	Precision	Recall	Precision	Recall
NM	Simulated				0.23	0.15	0.34	0.29	0.42	0.39
CB	Simulated			$\surd$	0.42	0.11	0.51	0.23	0.54	0.35
IB	Simulated	$\surd$			0.42	0.24	0.53	0.40	0.61	0.53
C&I	Simulated	$\surd$		$\surd$	0.57	0.24	0.66	0.40	0.69	0.55
IB (λ_0)	Simu		$\surd$		0.59	0.43	0.65	0.53	0.68	0.60
C&I (λ_0)	Simu		$\surd$	$\surd$	0.65	0.42	0.71	0.54	0.73	0.61
NM	Twitter				0.15	0.10	0.32	0.26	0.40	0.33
CB	Twitter			$\surd$	0.52	0.15	0.54	0.29	0.55	0.34
IB	Twitter	$\surd$			0.38	0.22	0.55	0.44	0.59	0.51
C&I	Twitter	$\surd$		$\surd$	0.59	0.21	0.65	0.44	0.70	0.53
