# Supplementary material for: Infectivity enhances prediction of viral cascades in Twitter
Source: PLoS One. 2019 Apr 17;14(4):e0214453. doi: 10.1371/journal.pone.0214453 (PMC6469756; doi:10.1371/journal.pone.0214453)
Supplement: S3 Table — (PDF) [file pone.0214453.s009.pdf]

Table 3. Logistic models of viral cascade prediction in Twitter data with estimated infectivity $\hat{\lambda}_0$. In all columns, variables such that $p < .05$ are highlighted with one asterisk, while variables such that $p < .01$ are highlighted with two asterisks. Standard errors are shown in parentheses.

Dependent	$\theta = 90\%$	$\theta = 80\%$	$\theta = 70\%$
Intercept	-5.3^{**} (1.2)	-4.0^{**} (0.85)	-3.1^{**} (0.70)
$\hat{\lambda}_0$	-0.58 (4.7)	5.43 (3.6)	8.0^{*} (3.2)
Early Adopters	0.03 (0.02)	-1.8×10^{-3} (0.01)	-3.4×10^{-3} (0.01)
neighbors	6.4×10^{-6} (6.5×10^{-5})	3.2×10^{-5} (4.9×10^{-5})	1.2×10^{-5} (4.3×10^{-5})
Infected Communities	-0.14^{**} (0.04)	-0.12^{**} (0.03)	-0.11^{**} (0.02)
H^r	2.1^{**} (0.63)	2.1^{**} (0.48)	1.9^{**} (0.40)
Intra-community	0.80 (1.1)	1.7^{*} (0.83)	1.7^{*} (0.69)
