# Supplementary material for: Infectivity enhances prediction of viral cascades in Twitter
Source: PLoS One. 2019 Apr 17;14(4):e0214453. doi: 10.1371/journal.pone.0214453 (PMC6469756; doi:10.1371/journal.pone.0214453)
Supplement: S4 Table — (PDF) [file pone.0214453.s010.pdf]

Table 5. Logistic models of viral cascade prediction in simulation data with true infectivity λ_0. In all columns, variables such that $p < .05$ are highlighted with one asterisk, while variables such that $p < .01$ are highlighted with two asterisks. Standard errors are shown in parentheses. Note that as we do not allow one node to retweet the same cascade more than once, the number of early adopters for the first 50 retweets is always 50 and therefore not included in the logistic regressions.

Dependent	$\theta = 90\%$	$\theta = 80\%$	$\theta = 70\%$
Intercept	-25.8^{**} (0.59)	-20.0^{**} (0.36)	-16.4^{**} (0.28)
λ_0	$1.3 \times 10^{3**}$ (31.3)	$1.00 \times 10^{3**}$ (18.9)	$8.4 \times 10^{2**}$ (14.2)
neighbors	$9.5 \times 10^{-4**}$ (2.1×10^{-5})	$9.5 \times 10^{-4**}$ (1.7×10^{-5})	$9.2 \times 10^{-4**}$ (1.5×10^{-5})
Infected Communities	0.04^{**} (0.02)	0.05^{**} (0.01)	0.06^{**} (0.01)
H^r	-0.85^{**} (0.15)	-0.84^{**} (0.12)	-0.86^{**} (0.10)
Intra-community	-0.02 (0.17)	-0.11 (0.13)	-0.13 (0.11)
