# Supplementary material for: Infectivity enhances prediction of viral cascades in Twitter
Source: PLoS One. 2019 Apr 17;14(4):e0214453. doi: 10.1371/journal.pone.0214453 (PMC6469756; doi:10.1371/journal.pone.0214453)
Supplement: S1 File — (PDF) [file pone.0214453.s012.pdf]

Supporting Information for:
Infectivity enhances prediction of viral cascades in Twitter

Weihua Li[1,2,3*], Skyler J. Cranmer[4], Zhiming Zheng[1], Peter J. Mucha[5]

1 LMIB, BDBC and School of Mathematics and Systems Science, Beihang University, Beijing 100191, China
2 Department of Computer Science, University College London, London WC1E 6EA, UK
3 Systemic Risk Centre, London School of Economics and Political Sciences, London WC2A 2AE, UK
4 Department of Political Science, The Ohio State University, Columbus, OH 43210, USA
5 Department of Mathematics, The University of North Carolina, Chapel Hill, NC 27599, USA

1 Empirical Twitter data

The empirical data we use in this paper, developed by and studied previously in Refs. [1] and [2], were sampled from Twitter between March 24, 2012 and April 25, 2012. New memes are defined as those with fewer than 20 tweets during the previous month, and only new memes that emerged during the observation time window were selected. The data contain the follower network with reciprocal following ties, the timeline hashtag data set of tweets generated by the users in the follower network, and the timeline hashtag data set of retweets with information of the retweeted user along with the user from whom the tweet originated.

Many studies have used hashtags as memes for exploring information diffusion processes in online social platforms [3, 4]. Displaying distribution statistics from empirical Twitter data for both hashtags (or memes) and retweet cascades in 1, we show that they are likely to be driven by different underlying dynamics. For example, under fitting a power-law distribution (without arguing about the validity of doing so) the exponents are fairly different: $\gamma = 1.9$ for hashtags and $\gamma = 2.3$ for cascades. Distributions with $\gamma < 2$ and $2 < \gamma < 3$ exhibit distinct statistical features: the first moment of a power-law distribution with $\gamma < 2$ is infinite, while it is finite for distributions with $\gamma > 2$, indicating that on average a hashtag appears in a huge number of tweets, while the size of a cascade is usually moderate. Viral hashtags can reach broader audience, have more extensive global impact, and are largely influenced by broadcasting. In contrast, retweet cascades are mostly spread by more immediate followers, have a more local impact, and are mainly transmitted via link contagion through personal ties in social networks.

2 Analysis of branching dynamics

We adopt the underlying network topology of Ref. 4, in the form of a directed social network such as Twitter, where there are N users represented by nodes in the system.

In our analysis we will regard $N \to \infty$. A randomly picked node has k followers with probability p_k, while it follows $\langle k \rangle$ others with $\langle k \rangle$ denoting the mean out-degree $\langle k \rangle = \sum_k k p_k$. The out-degree follows the power-law distribution with $p_k \propto k^{-\gamma}$. We set up an identical user memory length M for every individual. Only tweets within the Mth ranking can be seen and retweeted by the user. The ranking only accounts for the aging of tweets with new tweets always ranking higher than old ones.

We set the infectivity of the focal tweet as λ_0 and the average infectivity as $\langle \lambda_0 \rangle$ for all other tweets, which is the probability that a tweet will be chosen to be retweeted. For simplicity, the decay effect of tweets will not be considered initially here (but will be introduced further below). To measure the activity of tweet creation by users, we define the innovation rate as β, which is the probability that a user generates a brand new tweet. Throughout this section we consider a small time window Δt so that for any user at most one tweet will be created during this time. A user can also retweet old tweets on the screen with probabilities according to their infectivities. All of these tweets, whether innovated or retweeted, will appear on the screen of its followers, by overwriting existing tweets. To simplify the analysis, we assume that during updates any of the old existing tweets will be overwritten with the same probability. Thus if a user receives l new tweets from her followed nodes, we randomly pick l slots on her screen to clear out to write the new ones on.

If the focal tweet is tweeted by the user, then it will be cleared from her screen. The vacant slot will be filled in by a randomly chosen tweet whose infectivity is $\langle \lambda_0 \rangle$. At any time, as a user has $\langle k \rangle$ followed nodes, there will be on average $(\langle k \rangle + 1)\beta$ newly generated tweets and $\langle \lambda_0 \rangle \langle k \rangle M$ retweeted existing tweets in the next time step. If a user knows the focal tweet already, in other words she has the tweet on the screen, then the probability that the focal tweet will be overwritten in the next time step is

$$\rho = \begin{cases} 1, & \frac{\beta(\langle k \rangle+1)+\langle \lambda_0 \rangle \langle k \rangle M}{M} \geq 1, \\ \frac{\beta(\langle k \rangle+1)+\langle \lambda_0 \rangle \langle k \rangle M}{M}, & \frac{\beta(\langle k \rangle+1)+\langle \lambda_0 \rangle \langle k \rangle M}{M} < 1. \end{cases} \quad (1)$$

Throughout this paper we set parameters so that $\frac{\beta(\langle k \rangle+1)+\langle \lambda_0 \rangle \langle k \rangle M}{M} < 1$. Thus the overwriting probability is given by

$$\rho = \frac{\beta(\langle k \rangle + 1) + \langle \lambda_0 \rangle \langle k \rangle M}{M}. \quad (2)$$

The tweet we are interested in starts to spread from a randomly chosen root screen. Each retweet of the tweet adds 1 to the popularity. We denote $G(a, x)$ as the probability generating function (PGF) of the excess popularity distribution. At age a (i.e., at time $t + a$, where t is the birth time for the focal tweet), we define the PGF $H(a, x)$ for the popularity distribution of our focal tweet [4]

$$H(a, x) = \sum_n q_n(a) x^n, \quad (3)$$

where $q_n(a)$ is the probability that the tweet has been retweeted n times. It is also convenient to define the PGF $G^{(k)}(a, x)$ for the popularity distribution that the focal tweet originates from a root screen with out-degree k [5, 6]

$$G(a, x) = \sum_k p_k G^{(k)}(a, x), \quad (4)$$

and

$$H(a, x) = x G(a, x) f(G(a, x)). \quad (5)$$

For simplicity, we just focus on the model with only one node with the tweet on her screen at the initial stage. Now consider the focal tweet posted up for the first time on a

screen with out-degree k (call this screen $S1$), at time 0. We let $T(a)$ be the random variable for the number of tweets originated from a randomly picked node, and $T_k(a)$ the number of tweets originated from a node with degree k, at time a for the focal tweet. In other words, the tweet has age a at the observation time. At the next time step Δt, there will be three possible outcomes on screen $S1$ that contribute to the PGF $G^{(k)}(a,x)$:

(1) The tweet is retweeted, then removed from the screen $S1$, and shows up on the screens of all its k followers. This happens with probability $\lambda_0 \Delta t$, and the number of tweets under this scenario, denoted by $T_{k,1}(a)$, can be further rewritten by $T_{k,1}(a) = 1 + kT(a - \Delta t)$, as the degree of the followers are random. Note that at time Δt, the age will be $a - \Delta t$ at the observation time. This contributes $x[G(a-\Delta t, x)]^k$ to $G^{(k)}(a,x)$.

(2) The tweet is not retweeted but overwritten by other tweets appearing on screen $S1$ during this time period. This happens with probability $(1-\lambda_0\Delta t)\rho\Delta t = \rho\Delta t + o((\Delta t)^2)$, and the number of tweets for this outcome is $T_{k,2}(a) = 0$. This contributes 1 to $G^{(k)}(a,x)$.

(3) The tweet doesn't retweet, and it survives this period of time. This happens with probability $(1-\lambda_0\Delta t)(1-\rho\Delta t) = 1 - \lambda_0\Delta t - \rho\Delta t + o((\Delta t)^2)$, and $T_{k,3}(a) = T_k(a-\Delta t)$. Thus it contributes $G^{(k)}(a-\Delta t, x)$ to $G^{(k)}(a,x)$.

Putting (1), (2) and (3) together, as each outcome is independent and exclusive to the others, we have

$$\begin{aligned} G^{(k)}(a,x) = \lambda_0 \Delta t x [G(a-\Delta t, x)]^k + \rho \Delta t \\ +(1-\lambda_0\Delta t - \rho\Delta t) G^{(k)}(a-\Delta t, x), \end{aligned} \tag{6}$$

which is correct to first order in Δt. Regarding $\Delta t \to 0$ we arrive at

$$\frac{\partial G_{(k)}}{\partial a} = \lambda_0 x [G]^k + \rho - (\lambda_0 + \rho) G^{(k)}. \tag{7}$$

Multiplying p_k on both ends of equation (7) and summing over all k yields

$$\frac{\partial G}{\partial a} = \lambda_0 x f(G) + \rho - (\lambda_0 + \rho) G. \tag{8}$$

We now use this partial differential equation to find the mean popularity of the focal tweet at age a

$$m(a) \equiv \sum_{n=1}^{\infty} n q_n(a) = \frac{\partial H}{\partial x}(a,1) = 1 + (1 + \langle k \rangle) \frac{\partial G}{\partial x}(a,1). \tag{9}$$

Note that $G(a,1) = 1$, $f(1) = 1$, $f'(1) = \langle k \rangle$ and $m(0) = 1$. Differentiating equation (9) with respect to x we have

$$\frac{\mathrm{d}m}{\mathrm{d}a} = \lambda_0(1 + \langle k \rangle) + (\lambda_0 \langle k \rangle - \lambda_0 - \rho)(m - 1), \tag{10}$$

with $m(0) = 1$. We now make further observations about the infectivity λ_0 of the focal tweet.

The focal tweet has a constant infectivity during the entire diffusion process. We use $\tau = 1/(\rho - \lambda_0(\langle k \rangle - 1))$ to rewrite equation (10) to obtain the following result:

$$m(a) = \begin{cases} (2\lambda_0 + \rho)\tau + (1 - (2\lambda_0 + \rho)\tau)\exp\left(-\frac{a}{\tau}\right), & \lambda_0 \neq \frac{\rho}{\langle k \rangle - 1}, \\ 1 + \lambda_0(1 + \langle k \rangle)a, & \lambda_0 = \frac{\rho}{\langle k \rangle - 1}. \end{cases} \tag{11}$$

Here a spreading threshold appears with

$$\bar{\lambda_0} = \frac{\rho}{\langle k \rangle - 1} = \frac{\beta(\langle k \rangle + 1) + \langle \lambda_0 \rangle \langle k \rangle M}{M(\langle k \rangle - 1)}. \tag{12}$$

It shows that tweets with infectivity less than $\bar{\lambda_0}$ typically won't successfully spread out; they are likely to be forgotten before being retweeted even once.

3 Infectivity distribution

We propose a simple method to estimate the infectivity distribution of cascades from Twitter data. For a given retweet size S_i, the fraction of cascades with size $\geq S_i$ is calculated by $P(S \geq S_i) = N(S \geq S_i)/N_{cas}$, where $N(S \geq S_i)$ is the number of cascades with size $\geq S_i$. To associate the corresponding λ_{i0} with S_i, for a given decay factor α, we set the total time $t = 25$ in equation (4) in the main text and let $E(A_{i,t}|A_{i,t} \geq 1) = S_i$ to calculate the λ_{i0} on the right side. We assume that to reach a cascade of at least S_i retweets, the minimum infectivity is λ_{i0} calculated above, thus we could derive a complementary cumulative probability distribution of infectivity from Twitter data by $P(\lambda_0 \geq \lambda_{i0}) = P(S \geq S_i) = N(S \geq S_i)/N_{cas}$, which are the dot plots in red and orange in Fig. 4a. Meanwhile, we assume that a cascade with infectivity λ_{i0} will either not be retweeted at all due to initial fluctuations, or will reach a retweet size S_i determined by equation (4) in the main text. With a given set of μ, σ and $\lambda_{\max}$ in the truncated lognormal distribution, we could fit a complementary cumulative probability distribution by $P(\lambda_0 \geq \lambda_{i0}) = P(\lambda_{\max}) - P(\lambda_{i0})$, which is the blue curve in Fig. 4a. Therefore we can compare theoretical lognormal distribution to the corresponding distribution of Twitter retweet data, with our parameter setting matching the real data quite well.

Note that the lognormal parameters change if we reset the decay parameter α. In the main text we use $\alpha = 0.01$ with $\mu = \ln 0.0012$, $\sigma = \ln 2.4$ and $\lambda_{\max} = 0.017$. We show the true infectivity distribution used in simulations and the estimated infectivity distribution for cascades with at least 50 retweets in 2. The estimated infectivities of popular cascades is usually larger than the $\lambda_{\max}$ used in simulations. This suggests that our method overestimates the infectivities of popular cascades while underestimating that of not-retweeted cascades by an infectivity $\hat{\lambda_0} = 0$.

4 Simulation details

In our simulation model, we use one day as one time step, since our model requires that the rate of user activity is homogeneous across time steps (e.g., most people don't use Twitter after midnight). The simulation starts with each individual generating a cascade by innovation probability $\beta = 0.528$ at each time step, the infectivity of which is sampled from the truncated lognormal distribution $p^{\text{infectivity}}(\lambda_0)$ where $0 < \lambda_0 < \lambda_{\max}$. The cascade will then be seen by all of her followers. The attention length of each user (the maximum number of tweets within her attention) is set to $M = 43$. When new tweets appear, the oldest ones will be forgotten by the user. At each time step, a user can either post new cascades, or retweet any cascade received from others according to its infectivity. The infectivity of cascade i will decrease according to the imposed decay factor as

$$\lambda_i(t) = \lambda_{i0} e^{-\alpha(t - t_{i0})}. \tag{13}$$

In the beginning of the simulation, no retweets are in the system. After around 10 time steps the number of retweets generated by the users within each time step will become

stable. Therefore we take the first 100 time steps of the simulation as the "burn–in" stage, and collect simulated retweet data from the 101st to the 133rd time step.

5 Other model specifications

We present simulation results based on other parameter specifications, as a robustness test for models discussed in the main text. First, fixing values of other parameters, we change the decay factor α to new values 0.001 and 0.02, and the $\lambda_{\max}$ in lognormal distribution to 0.0158 and 0.018, respectively. The fit to equation (6) in the main text is shown in 4, suggesting that when changing the decay parameter we can still fit well to the lognormal distribution of infectivities of cascades.

We try to replicate our simulation model in several networks. In 3 we show the degree distribution of some of the candidate networks, including the Twitter reciprocal network, the Barabàsi-Albert network [7], and synthetic networks [8] with exponents $\gamma = 2.5$ and $\gamma = 2.8$. More detailed statistics of these networks are presented in 1.

To test whether the decay effect of infectivity affects the results of our model, we also run simulations with different parameter settings: When $\alpha = 0.001$, we fix $\mu = \ln 0.0012$, $\sigma = \ln 2.4$ and change $\lambda_{\max}$ to 0.0158; When $\alpha = 0.02$, we also fix $\mu = \ln 0.0012$, $\sigma = \ln 2.4$ but change $\lambda_{\max}$ to 0.018.

With the above new parameter settings, we run simulations on the Twitter network, reconfigured random network that preserves the degree distribution of the empirical Twitter network, and a synthetic network [8] with power-law exponent $\gamma = 2.8$ to verify results in the main text. The selected networks appear to be reasonable power-law approximations to the degree distribution of the empirical Twitter network, and the simulation results in these networks match the empirical retweet distribution well (5). These findings show that our model is not sensitive to specific network topology with similar degrees of heterogeneity.

6 Prediction and random forests models

Since our simulation model does not allow an individual to retweet the same cascade repeatedly, the "user entropy" from Ref. 1 is the same as retweet entropy H^r and therefore not discussed in this paper. In addition, Weng et al. defined four baseline models of information spread in Ref. 1: the random sampling model (M1); the simple cascade model (M2) that accounts only for the network structure; the social reinforcement model (M3) that chooses the user with maximum number of infected neighbors to adopt the cascade; and the homophily model (M4), which assumes that only neighbors in the same community can retweet the cascade.

The detailed definition of baseline models are as follows: For a given cascade, M1 randomly samples the same number of retweets as in the real data. M2 randomly selects a user, and at each time step with probability 0.85, we randomly select one of its neighbors to retweet, or with probability 0.15 the process restarts from a new user. Comparing to M1, M2 takes the network structure into account. The cascade in M3 is generated similarly to M2 but at each time step the user with the maximum number of infected neighbors retweet the cascade. M3 accounts for the social reinforcement effect. M4 simulates in the same way as in M2 but at each step, only neighbors in the same community can retweet the cascade, which accounts for homophily effect [1].

As a direct comparison between our simulation model and the baseline models, in 6 we present the g and H^r statistics of cascade diffusion scaled by that of M1 for the other baseline models and our simulation model. Though our model assumptions do not account for community structures, it nevertheless outperforms other baseline models.

We use the *InfoMap* algorithm for community detection in the Twitter follower network [9]. We run random forests models by using 10-fold cross validation. Variables used in random forests models include:

Two null model predictors: The number of distinct users: the number of distinct retweet users in the first 50 retweets of a given cascade; The total number of neighbors of early retweet users: for each cascade, sum up the number of users who retweeted the first 50 retweets [1];

Three community–based predictors: The number of infected communities: the number of distinct communities that has at least one user who retweeted in the first 50 retweets; Retweet entropy H^r: the entropy based on how retweet users of a cascade are distributed across different communities. It is computed based on the first 50 retweets; Fraction of intra–community user interactions: count pair-wise user interactions for a given cascade, and compute the proportion that occur between users in the same community. For retweet data, as we only know the original user who posted the retweet and the users who retweeted it, this measure becomes the fraction of first 50 retweet users who are in the same community as the original user [1];

Estimated infectivity $\hat{\lambda_0}$: we use equation (2) in the main text to estimate the infectivity of a given cascade based on the first 50 retweets. Note that this should be interpreted as the infectivity without a decay parameter. To numerically obtain $\hat{\lambda_0}$ we start from $\hat{\lambda_0} = 0$ and increase by 0.0001 to get the value that makes the right side of equation (2) closest to 50.

The random forests results are presented in Fig. 3 and 2. Note that in 2 we also run models using the true infectivity λ_0 on simulation models. This improves the prediction and recall rates substantially compared to the random forests models using the estimated infectivity $\hat{\lambda_0}$, suggesting that an improved estimation method for infectivity could be a key factor for the improved prediction of viral cascades in Twitter.

We also run logistic regressions with the predictors used in random forests models in 3, 4, and 5. These results are discussed in detail in the main text of the paper.

References

1. Weng L, Menczer F, Ahn YY. Virality Prediction and Community Structure in Social Networks. Sci Rep. 2013;3.
2. Weng L, Menczer F, Ahn YY. Predicting Successful Memes Using Network and Community Structure. ICWSM. 2014;.
3. Weng L, Flammini A, Vespignani A, Menczer F. Competition among memes in a world with limited attention. Sci Rep. 2012;2.
4. Gleeson JP, Ward JA, O'sullivan KP, Lee WT. Competition-induced criticality in a model of meme popularity. Phys Rev Lett. 2014;112(4):048701.
5. Newman ME, Strogatz SH, Watts DJ. Random graphs with arbitrary degree distributions and their applications. Phys Rev E. 2001;64(2):026118.
6. Wilf HS. Generatingfunctionology. Elsevier; 2013.
7. Barabási AL, Albert R. Emergence of scaling in random networks. Science. 1999;286(5439):509–512.
8. Goh KI, Kahng B, Kim D. Universal behavior of load distribution in scale-free networks. Phys Rev Lett. 2001;87(27):278701.

9. Rosvall M, Bergstrom CT. Maps of random walks on complex networks reveal community structure. Proc Natl Acad Sci USA. 2008;105(4):1118.
